# Isometric force production parameters during normal and experimental low back pain conditions

**DOI:** 10.1186/1471-2474-6-6

**Published:** 2005-02-09

**Authors:** Martin Descarreaux, Jean-Sébastien Blouin, Normand Teasdale

**Affiliations:** 1 Faculté de Médecine, Division de Kinésiologie, U. Laval, Canada; 2 Département de Chiropratique, Université du Québec à Trois-Rivières, Canada

## Abstract

**Background:**

The control of force and its between-trial variability are often taken as critical determinants of motor performance. Subjects performed isometric trunk flexion and extension forces without and with experiment pain to examine if pain yields changes in the control of trunk forces. The objective of this study is to determine if experimental low back pain modifies trunk isometric force production.

**Methods:**

Ten control subjects participated in this study. They were required to exert 50 and 75% of their isometric maximal trunk flexion and extension torque. In a learning phase preceding the non painful and painful trials, visual and verbal feedbacks were provided. Then, subjects were asked to perform 10 trials without any feedback. Time to peak torque, time to peak torque variability, peak torque variability as well as constant and absolute error in peak torque were calculated. Time to peak and peak dF/dt were computed to determine if the first peak of dF/dt could predict the peak torque achieved.

**Results:**

Absolute and constant errors were higher in the presence of a painful electrical stimulation. Furthermore, peak torque variability for the higher level of force was increased with in the presence of experimental pain. The linear regressions between peak dF/dt, time to peak dF/dt and peak torque were similar for both conditions. Experimental low back pain yielded increased absolute and constant errors as well as a greater peak torque variability for the higher levels of force. The control strategy, however, remained the same between the non painful and painful condition. Cutaneous pain affects some isometric force production parameters but modifications of motor control strategies are not implemented spontaneously.

**Conclusions:**

It is hypothesized that adaptation of motor strategies to low back pain is implemented gradually over time. This would enable LBP patients to perform their daily tasks with presumably less pain and more accuracy.

## Background

There are many possible explanations for the origin and consequences of acute and chronic low back pain but the transition from acute to chronic low back conditions needs to be clarified [1,2]. There is a number of recent evidences suggesting that chronic LBP patients exhibit deficits in proprioception and trunk motor control. For example, changes in postural control[3], delayed muscle responses to sudden trunk loading[4], increased trunk movement detection threshold[5] and increased repositioning errors in patients with LBP have all been reported[6,7].

For example, Oddsson et al.[8] used spectral parameters of the surface electromyographic (EMG) signal from lumbar back muscles assessed during a fatiguing isometric contraction to classify LBP and healthy subjects. They observed more activation imbalances in chronic LBP subjects and proposed that these changes would eventually become «normal» behavior for the chronic LBP individuals[8,9]. They suggested that changes in the trunk muscular activity could result from subtle postural adjustments that were developed during the acute phase to avoid pain. These observed changes imply a certain level of adaptation to the initial acute pain. Motor control in chronic subjects can be influenced by the presence of chronic pain but also by other phenomenon like type II fiber atrophy, degenerative changes and decreased trunk muscle force and endurance[10].

Clinical studies of chronic LBP patients involve heterogeneous populations and the effect of chronic pain cannot be differentiated from other degenerative and functional changes occurring in the lumbar spine. Experimentally induced LBP eliminates some of these uncertainties and could allow an examination of the effect of pain per se[11]. To examine the effect of experimental pain on the sensori-motor control of the lumbar spine, two different protocols have been used in the past: (1) lumbar cutaneous pain induced by electrical or mechanical stimulation[11] and (2) deep lumbar pain induced by saline injection of lumbar muscles[11,12]. Zedka et al.[11] noted increased stretch reflex responses in the presence of cutaneous electrical and mechanical stimulations. They also noted an increase in EMG amplitude (during extension) as well as several changes in the motor patterns (trunk velocity and range of motion) in presence of muscle pain induced by a saline injection. Hodges and is colleagues recently demonstrated that feedforward recruitment of trunk muscles is altered in presence of experimental and clinical LBP[13,14]. In a postural task where the subjects were asked to rapidly flex the upper limb, they noted delayed transversus abdominis muscles activation in the presence of experimental pain[14] or chronic low back pain[13]. They concluded that some changes in motor control that occur in LBP patients (experimental or chronic) may be caused by pain. These modifications do not resolve spontaneously with alleviation of symptoms since the effect was still observed after a 10-min delay.

In a previous study, we have observed that two different control strategies were used by chronic LBP subjects to produce accurate trunk isometric forces[15]. One subgroup of LBP subjects used an open-loop control strategy similar to that used by healthy control subjects whereas a second subgroup of subjects used a less open-loop control strategy characterized in part by a longer time to peak force. Both LBP subgroups, however, were able to produce isometric trunk forces as accurately as the healthy subjects.

The aim of the present study was to evaluate if a painful stimulation, induced by cutaneous electrical stimulation, would spontaneously yield a change in the control strategy or the variability of trunk isometric force production. To explore whether isometric trunk forces are similarly programmed with and without experimental pain, we used the model proposed by Gordon and Ghez[16]. If motor planning is affected by lumbar cutaneous pain, a more closed-loop mode of control characterized by an increased time-to-peak force and a lack of relationship between the peak of dF/dt and the peak force should be observed. On the other hand, the absence of such a change in the mode of control could yield a more variable force production resulting from sensory and motor effects of pain on the motor response.

## Methods

Force production parameters were measured in 10 healthy subjects with no history of chronic or recurrent LBP (10 men, age: 25.9 years). Each subject gave their written inform consent and the study was approved by the local ethics committee. All subjects were university students. Force data (torque) were obtained from an isometric testing apparatus (Loredan Biomedical, West Sacramento, USA) and recorded at a sampling rate of 500 Hz. Torque data were digitally filtered with a seventh-order dual pass Butterworth filter (7 Hz low pass cut-off frequency). The first time derivative of torque was calculated using a finite difference algorithm (window 25 ms).

Superficial pain was elicited by electrical stimulation of the skin over the spinous process of L3 using bipolar surface Ag-AgCl electrodes (Beckman electrodes, 1 cm diameter). This site of stimulation was chosen to ensure that no direct muscular activation could result from the electrical stimulus. The range of voltage used during our experiment was 135–140 Volts. This stimulation created a focal cutaneous painful stimulus with very limited current spread. The technique for inducing the pain stimulation was inspired from the work of Arendt-Nielsen et al.[17]. Each stimulus consisted of a standard 3-second constant-voltage pulse train of 1-ms pulses delivered at 10 Hz (ISI = 100 ms; S88 Grass stimulator with SIU8T constant voltage isolation unit, USA). The amplitude used for the stimulation was determined when the subjects quoted the pain intensity of the stimulation between 7.5 and 8.5 on a 0–10 scale. The intensity of pain was monitored throughout the experiment and adjusted accordingly to ensure a constant pain level.

Testing was done in a neutral standing posture (no trunk flexion or extension). First, maximal isometric flexion and extension torques of trunk muscles were collected. The higher torque value obtained in three consecutive 4-second trials was used as the reference for maximal voluntary contraction. Then, four experimental conditions of trunk torque production were evaluated without and with experimental pain: 50 and 75% of the maximal isometric torque in both extension and flexion. Conditions were presented by block with the order of presentation being randomized across subjects. For each condition, trials without pain were presented first followed by the trials with pain. For each trial, subjects were instructed to produce a trunk isometric force as quickly as possible following an auditory signal. They were encouraged to produce a single impulse ("shoot and release") and to make no attempt at correcting the force once the contraction was initiated. For each condition, a learning phase was provided. During this phase, after each trial, subjects were given visual accuracy feedback through an oscilloscope located in front of them. Subjects were specifically asked to produce peak torques that were within 10% of the goal target. This learning phase stopped when five consecutive contractions were within the 10% margin. Following these learning trials, subjects performed 10 consecutive trials without any visual feedback. The pain condition followed. A second learning sequence with feedbacks and without pain was given to the subjects. This procedure was used to insure that no differences between the control and pain conditions would reflect a pain effect and not a loss of calibration after a block of trials without pain. Hence, if any differences between the control and pain conditions were found, these would reflect a pain effect and not a learning effect. For the pain trials, the stimulation was initiated 0.5 sec before an auditory tone indicating the subjects to initiate the contraction. All dependent variables were derived from the behavior observed for the 10 trials without feedback without and with the experimental pain.

For each experimental trial, the onset of torque and peak torque were determined. Using this information, time to peak torque, time to peak torque variability, peak torque variability as well as the constant and absolute error in peak torque were calculated for each condition. Constant error represents the positive or negative difference between the peak torque reached and the target torque. Absolute error in peak torque represents the positive difference between the reached peak torque and the goal peak torque whereas time to peak represents the period of time between the beginning of rising torque and the maximal torque obtained in the trial. Peak dF/dt was also computed to examine if the first peak of dF/dt could predict the peak torque achieved. Linear regressions were calculated for each subject and a high r^2^, indicating that the first peak of dF/dt could predict the peak torque, was taken as an indication of a preprogrammed or open-loop mode of control[16].

## Results

On average, the maximal voluntary contraction in flexion and extension were 236.2 Nm and 346.5 Nm, respectively. Table 1 presents a summary of the statistical analyses for all dependant variables. The ANOVA for absolute errors yielded a main effect of Pain. Absolute errors were higher in the painful condition than in the normal condition (30.7 Nm vs 23.9 Nm respectively; F1-9 = 8.29, *p *= 0.018). The main effects of Direction, Force level and all interactions were not significant (ps > 0.05). Similar observations were made for the constant errors as the ANOVA yielded a main effect of Pain (F1-9 = 6.22, *p *= 0.035). The main effects of Direction, Force level and all interactions also were not significant (ps > 0.05). The painful stimulus yielded increased constant and absolute errors indicating that subjects, on average, overshot the target by 25.9 Nm (13.9 Nm for the normal condition).

**Table 1:** Statistical analyses for all dependant variables.

	**Pain (P)**	**Direction (D)**	**Force Level (F)**	**P × D**	**D × F**	**P × F**
Time to peak force	*F *= 0.017 *p *= 0.901	*F *= 1.094 *p *= 0.323	*F *= 0.541 *p *= 0.481	*F *= 1.011 *p *= 0.341	*F *= 2.009 *p *= 0.190	*F *= 2.628 *p *= 0.139

Time to peak force variability	*F *= 8.763 *p *= 0.016*	*F *= 0.083 *p *= 0.779	*F *= 0.005 *p *= 0.943	*F *= 0.115 *p *= 0.742	*F *= 3.904 *p *= 0.080	*F *= 0.028 *p *= 0.870

Peak force variability	*F *= 7.756 *p *= 0.021*	*F *= 1.183 *p *= 0.209	*F *= 0.487 *p *= 0.503	*F *= 0.032 *p *= 0.861	*F *= 0.096 *p *= 0.764	*F *= 8.047 *p *= 0.020*

Constant error	*F *= 6.222 *p *= 0.034*	*F *= 0.530 *p *= 0.485	*F *= 5.02 *p *= 0.052	*F *= 5.018 *p *= 0.053	*F *= 0.273 *p *= 0.614	*F *= 0.027 *p *= 0.873

Absolute error	*F *= 8.289 *p *= 0.018*	*F *= 1.1 *p *= 0.482	*F *= 0.537 *p *= 0.482	*F *= 0.909 *p *= 0.365	*F *= 2.222 *p *= 0.170	*F *= 1.158 *p *= 0.310

*p < 0.05

**Figure 1:**
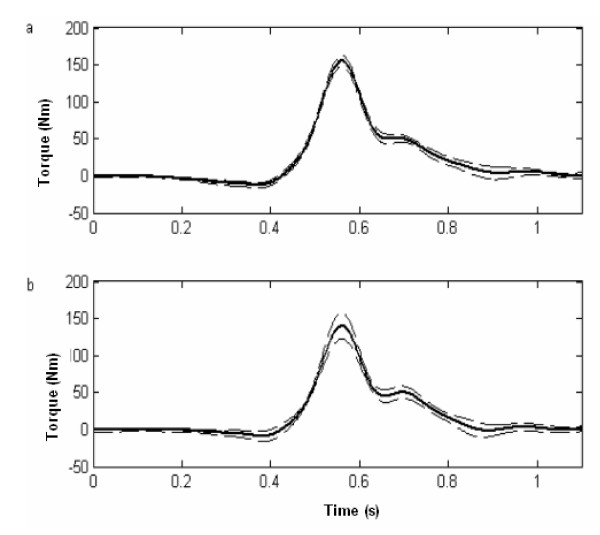
Typical torque-time curves illustrating the mean (SD is represented by the dashed line) of ten consecutive flexion trials (without feedback) in the control condition. (b) Typical torque-time curves illustrating the mean (SD is represented by the dashed line) of ten consecutive flexion trials (without feedback) in the experimental pain condition.

Figure 1 illustrates, for one subject, the mean and variability of ten consecutive flexion trials (50% of maximal flexion torque) without and with pain. With pain, the torque-time curves exhibit greater variability around the peak.

**Figure 2:**
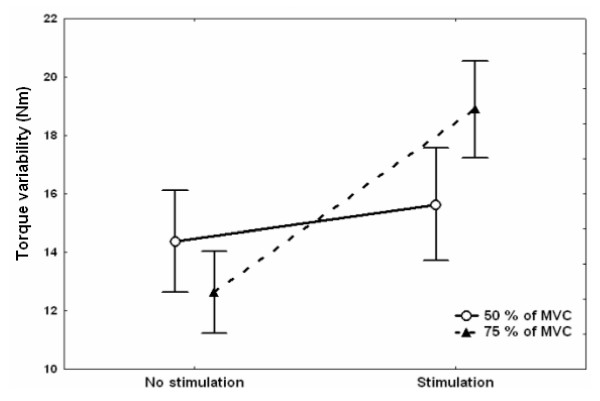
Mean (SD) peak torque variability with and without electrical stimulation for both levels of force.

Figure 2 illustrates peak torque variability with and without experimental pain for both levels of force. The ANOVA for peak torque variability showed a significant main effect of Pain (F 1,9  = 7.76, *p *= 0.021) and an interaction of Pain × Force (F 1,9  =  8.05, *p *= 0.020) but no main effect of Force (ps > 0.05). A decomposition of the interaction showed that peak torque variability increased with the painful stimulation only for the higher level of force. For the higher level of force, the variability was 12.6 Nm in the control condition and 18.6 Nm in the presence of electrical stimulation (Tukey: *p *= 0.027). For the lower level of force, peak torque variability was similar for both conditions (15.0 Nm; p > 0.05).

The average time to peak torque was not affected by the lumbar electrical stimulation. On average, the time to peak torque was 240 ms. The main effects of Pain, Direction, Force level and all interactions were not significant (*p*s > 0.05). The time to peak torque variability, however, was increased in the painful condition than in the normal condition (81 ms vs 58 ms respectively; F1-9 = 8.76, *p *= 0.016). Again, the main effects of Direction, Force level and all interactions were not significant (ps > 0.05).

The dF/dt curves for both conditions are characterized by a single peak in the first phase of isometric force production. On average, peak dF/dt explained 73.5 and 74.3 percent of the variance observed in peak torque for the normal and the painful conditions, respectively (p > 0.05). This suggests a similar control strategy for both the normal and the painful conditions.

## Discussion

The presence of an experimental cutaneous lumbar pain altered the production of isometric trunk forces in various ways. Specifically, when exposed to the painful cutaneous electrical stimulation, subjects showed greater absolute and constant errors in isometric trunk torque production. The effects of a lumbar cutaneous painful stimulation on the isometric trunk force production yielded an overestimation of the learned level of force to be performed in both flexion and extension. This observation argues against a specific modification of the trunk flexor or extensor motoneuronal pool following the painful stimulation. Rather it appears that, independently of the direction of the force production required, the pain stimulation yielded an increased excitability of the agonist motor pathways. Our results, however, cannot discriminate if these modifications occurred at the programming stage or at the execution stage (upper and lower motorneurons). All of these mechanisms have previously been suggested to explain the modifications induced by experimental pain[11,14,18]. Across the painful trials, subjects also showed greater torque variability for the higher level of forces. Time to peak torque variability was also greater with than without pain. Linear regressions between peak dF/dt and peak torque however, were similar in both conditions indicating that the subjects used a similar strategy force control strategy without and with pain. In a previous study, chronic low back pain patients demonstrated longer time to peak values suggesting a shift from an open loop strategy of control to a more close loop strategy of control[15]. Results of the present experiment suggest that, in the presence of experimental cutaneous pain, subjects maintained an open loop control strategy to perform the task. Such an absence of modification in the control strategy could be specifically related to the task used in the present experiment. Subjects were explicitly told to perform the isometric force production as quickly and accurately as possible.

Numerous authors have showed that chronic LBP patients exhibit deficits in proprioception and trunk motor control [3-7]. The link between persistent pain and subsequent adaptations to low back symptoms remains unclear although some hypotheses have been formulated. Lund et al. first proposed a pain adaptation model that could occur in the presence of persistent pain[19]. This adaptation is characterized by an increased motoneuron output when the muscle is acting as an antagonist and by a decreased motoneuron output when the muscle is acting as an agonist. Luoto et al., throughout a series of study, have shown that motor control deficits observed in chronic LBP subjects can be, at least in part, due to impairment in central processing. According to the authors, pain would consist of an irrelevant sensory input that cannot be ignored but that is hampering central processing[20,21]. Even though pain probably causes peripheral adaptations, central impairment must also be considered. Oddsson and his colleagues[9] suggested that chronic low back patients, for whom acute pain reactions are no longer present, could develop a new strategy (postural adjustments) to avoid the sensation of pain. Although experimentally induced pain is different from clinical pain, some authors reported motor changes similar to those observed in LBP patients when inducing experimental pain in control subjects[14]. Hodges et al. observed that experimental and recurrent low back pain induced similar delays in transversus abdominis activation[4,13,14]. Zedka et al[11] observed a decrease in velocity and range of trunk motion after saline injection of lumbar muscles similar to those observed in patients with low back pain. They also demonstrated an increased excitability in the long latency lumbar response after a painful cutaneous electrical stimulation. These changes were attributed to the interactions between nociceptives afferents and motor neuron pool excitability[17]. Overall, it appears that both clinical and experimental low back pain can influence trunk muscle activations suggesting that the sole presence of pain is detrimental to motor performance.

The painful stimulus used in the present experiment consisted of trains of 1 ms electrical pulses applied to the spinous process of the third lumbar vertebra. Although the experimenter could not observe nor palpate any muscular contractions and the subjects did not report any involuntary muscular contractions, the possibility exists that spreading of the current over the surrounding tissues activated the neuromuscular junction of the nearby lumbar paraspinal muscles. Consequently, imperceptible muscular contractions could have occurred, particularly at the high level of Voltage used to elicit the perception of pain in our subjects. Muscular activation of the paraspinal muscles due to the current spreading while performing an isometric force reproduction task could certainly lead to a deterioration of the subjects' performance (both the accuracy and variability) under the experimental pain condition. However, the overestimation of the learned level of force was observed in both flexion and extension – whereas the painful stimulation was always applied to the spinous process (presumably biasing only the paraspinal muscles) – argues against this factor playing a critical role in the findings reported in the present manuscript. Further work, however, is needed to quantify the performance of healthy subjects performing an isometric force reproduction task using a different experimental pain protocol (e.g. muscle saline injection).

In a previous study, we observed that some chronic LBP patients, compared to healthy control subjects, adopted a more close loop control strategy of trunk isometric force production to maintain a particular level of performance[15]. Both experiments used a similar protocol of isometric force production but the present results failed to reveal any changes in the control strategy adopted by the subjects. On the other hand, in the presence of experimental cutaneous pain, a less accurate isometric force production was observed. Therefore our results suggest that, even if some modifications occurred directly in the presence of pain[11,14], adaptation to low back pain and modifications of motor control strategies are not implemented spontaneously. It seems that the modification in control strategy observed for chronic LBP subjects could be an adaptation to limit the variability of force production. For control subjects, the "rise time regulation" strategy or variations thereof have been suggested to help in reducing response variability[16,22,23]. Also, it has been suggested that, in the presence of persistent experimental or chronic low back pain, subjects need to adapt their motor control strategies in order to limit exacerbation of pain symptoms[9,19]. Whether chronic LBP subjects adopt a new control strategy to limit their pain symptoms or to minimize their force production errors remains to be determined.

## Conclusion

The present data indicate that trunk isometric force production can be affected by experimental cutaneous low back pain. While the motor control strategy remained the same between the non painful and painful condition, subjects showed less accuracy and more variability in the painful condition. Experimental cutaneous low back pain is different from deep tissue pain and the observed changes. This precludes any generalization to acute low back pain. It is hypothesized, however, that adaptation of motor strategies to low back pain is implemented gradually over time. This would enable LBP patients to perform their daily tasks with presumably less pain and more accuracy.

## Competing interests

The author(s) declare that they have no competing interests.

## Authors' contributions

MD and JS performed all the testing and data analyses. NT acted as the thesis director of MD and JS and participated in the design and coordination of the study. All authors read and approved the final manuscript.

## Pre-publication history

The pre-publication history for this paper can be accessed here:


